# Direct Comparative Analysis of a Pharmacogenomics Panel with PacBio Hifi^®^ Long-Read and Illumina Short-Read Sequencing

**DOI:** 10.3390/jpm13121655

**Published:** 2023-11-27

**Authors:** David Barthélémy, Elodie Belmonte, Laurie Di Pilla, Claire Bardel, Eve Duport, Veronique Gautier, Léa Payen

**Affiliations:** 1 Institut of Pharmaceutical and Biological Sciences of Lyon, Claude Bernard Lyon I, 69373 Lyon, France; david.barthelemy@chu-lyon.fr (D.B.); claire.bardel@chu-lyon.fr (C.B.); 2 Department of Biochemistry and Molecular Biology, Lyon-Sud Hospital, Hospices Civils de Lyon, Réseau Francophone de Pharmacogénétique (RNPGx), 69495 Pierre-Bénite, France; laurie.di-pilla@chu-lyon.fr (L.D.P.); eve.duport@chu-lyon.fr (E.D.); 3 Center for Innovation in Cancerology of Lyon (CICLY) EA 3738, Faculty of Medicine and Maieutic Lyon Sud, Claude Bernard University Lyon I, 69921 Oullins, France; 4 Plateforme Génotypage et Séquençage en Auvergne (GENTYANE) UMR 1095 Génétique, Diversité Ecophysiologie des Céréales INRAE, Université Clermont Auvergne, 63100 Clermont Ferrand, France; elodie.belmonte@inrae.fr (E.B.); veronique.gautier@inrae.fr (V.G.); 5 Department of Bioinformatics, Hospices Civils de Lyon, 69008 Lyon, France

**Keywords:** long-read sequencing (LSR), short-read sequencing (SRS), pharmacogenetics, NGS, haplotyping, phasing

## Abstract

Background: Pharmacogenetics (PGx) aims to determine genetic signatures that can be used in clinical settings to individualize treatment for each patient, including anti-cancer drugs, anti-psychotics, and painkillers. Taken together, a better understanding of the impacts of genetic variants on the corresponding protein function or expression permits the prediction of the pharmacological response: responders, non-responders, and those with adverse drug reactions (ADRs). Objective: This work provides a comparison between innovative long-read sequencing (LRS) and short-read sequencing (SRS) techniques. Methods and Materials: The gene panel captured using PacBio HiFi^®^ sequencing was tested on thirteen clinical samples on GENTYANE’s platform. SRS, using a comprehensive pharmacogenetics panel, was performed in routine settings at the Civil Hospitals of Lyon. We focused on complex regions analysis, including copy number variations (CNVs), structural variants, repeated regions, and phasing-haplotyping for three key pharmacogenes: *CYP2D6*, *UGT1A1,* and *NAT2*. Results: Variants and the corresponding expected star (*) alleles were reported. Although only 38.4% concordance was found for haplotype determination and 61.5% for diplotype, this did not affect the metabolism scoring. A better accuracy of LRS was obtained for the detection of the CYP2D6*5 haplotype in the presence of the duplicated wild-type CYP2D6*2 form. A total concordance was performed for *UGT1A1* TA repeat detection. Direct phasing using the LRS approach allowed us to correct certain *NAT2* profiles. Conclusions: Combining an optimized variant-calling pipeline and with direct phasing analysis, LRS is a robust technique for PGx analysis that can minimize the risk of mis-haplotyping.

## 1. Introduction

Pharmacogenetics was initially limited to the analysis of drug metabolism enzymes, including drug transporters, cytochromes, and glucuronidation proteins, that can modify the drug pharmacokinetics (PK) parameters. Key PK genes are principally those encoding drug metabolizing (phase I and phase II) enzymes, drug transporters, and molecular targets with drug interactions. Pharmacogenetics studies the prediction of functional consequences of genomic variations such as the 2–3 million single nucleotide variants (SNVs), small insertions/deletions (InDels), and structural modifications (hybrids, allele duplication, or deletion) in the human genome. Currently, 201 clinical guideline annotations are listed in the Pharmacogenomics Knowledgebase (PharmGKB) [1]. This database integrates information from several academic societies including the Dutch Pharmacogenetics Working Group (DPWG, knmp.nl) and the Clinical Pharmacogenetics Implementation Consortium (CPIC, cpicpgx.org) [2,3]. These clinical annotations provide information about variant-drug pairs based primarily on variant annotations and incorporating variant-specific information for prescription guidance based on clinical guidelines and FDA-approved drug labels, when available [1].

In the present study, we focused on three major actors: CYP2D6, UGT1A1, and NAT2. The cytochrome P450 2D6 (*CYP2D6)* is a highly polymorphic gene coding for CYP2D6. Patients with a CYP2D6 phenotype (ultra-metabolizer, UM, or poor-metabolizer, PM) have a higher risk of modified toxicity (e.g., tramadol, codeine) or modified efficacy (e.g., tamoxifen) depending on whether a drug is bioactivated or metabolized by CYP2D6 [4,5,6]. Due to a complex genomic architecture including CNVs and hybrids of the *CYP2D6-CYP2D7* gene with homologous regions, having cutting-edge molecular tools is essential to enable effective biological analysis leading to individualized treatment [7]. Uridine diphosphate glucuronosyltransferase 1A1 (*UGT1A1)* is a gene comprising of a repeated nucleotide sequence of TA repeats in its promoter (Hg19/GRCh37 chr2:23466879-23466881). The TA repeated sequence is subject to a high degree of polymorphism directly affecting the expression level of UGT1A1. Patients diagnosed with Gilbert syndrome (UGT1A1*28) may be predisposed to potential ADRs under chemotherapy (e.g., irinotecan) [8,9]. The resulting phenotype is a reduced capacity of substrate glucuronidation by UGT1A1. Slow acetylator patients treated with the anti-tuberculosis drug isoniazid experienced ADRs due to the presence of specific variations in the N-acetyl-transferase 2 (*NAT2*) gene. Statistical and computational methods are required to infer the *cis-* and *trans-* haplotype configurations [10,11] as clinical variants are located on different exons. Clinical diplotypes derived from these statistically inferred haplotypes will determine the patient’s acetylation profile for the phase II enzyme NAT2 [12].

Currently, complex genomic region analysis coding PK genes is performed using the NGS-SRS technique followed by statistical phasing. Nevertheless, this only provides a probabilistic result [13]. SRS is subject to inherent limitations due to its short-read fragmentation (~350 pb with NEXTseq500 Illumina technology) and possible alignment errors in highly homologous genomics regions. These “dead zone regions” represent about 17% of the reference genome, including several key pharmacogenes like *CYP2D6* and *UGT1A1* [14]. To overcome this limit, in collaboration with Sophia Genetics, we set-up bioinformatics tools that consider only the reads bearing exclusive variants for the haplotyping of *CYP2D6* and its discrimination from *pCYP2D7*. In parallel, LRS combined with direct phasing allows for the amplification of long fragments (~1–5k bp) with a read depth of 15–30× coverage, overcoming this *cis-* and *trans-*determination limitation [15]. Amongst all the LRS techniques, PacBio SMRT^®^ and Oxford Nanopore^®^ are both third-generation HTS technologies. First-generation LRS techniques are characterized by a reduced read accuracy (75–99%) with high raw-read error rates (~10%) compared to SRS (<1%) [10,16,17]. HiFi sequencing is an evolution of the PacBio technology, allowing enhanced read accuracy from noisy individual subreads. HiFi reads are derived from a consensus sequence from multiple passes of a single-molecule real-time (SMRT) [18,19].

Here, the aim was to perform a retrospective comparison between HiFi^®^ LRS using a Twist Bioscience hybrid capture panel and Illumina^®^ SRS, using a comprehensive in-house panel for complex genomic regions analysis. We focused on three complex pharmacogenes: *CYP2D6*, *UGT1A1,* and *NAT2,* for which we have a real need in clinical settings. A comparable performance was found using SRS and LRS methods including SNVs, CNVs, and repeated sequences for genes covered by both panels. Direct phasing was performed after LRS for *NAT2* haplotyping and improved the acetylation status prediction.

## 2. Materials and Methods

### 2.1. Patient Cohort

Physicians informed patients or legal guardians and they signed a consent form for genomics analysis for pharmacogenetics. Whole blood samples were collected from patients during the consultation at the psychiatric unit of Vinatier Hospital (Lyon, France). Vinatier Hospital is a well-known expert center for multiple psychiatric pathologies. This hospital serves a diverse patient population presenting with a wide range of conditions, many of which are treated with pharmacotherapies that are influenced by patient-specific pharmacogenomic profiles. This makes it an ideal setting for a study focused on clinical applications. By concentrating our study within this expert center, we have been able to draw from a well-characterized and clinically relevant population. According to current French clinical practice, samples were anonymized before molecular analysis was carried out by the certified South Lyon Hospital’s Laboratory of Biochemistry and Molecular Biology. DNA was extracted within 2–3 weeks using genomic DNA (gDNA) blood extraction (Promega, Madisson, WI, USA, Ref. A6030). The gDNA was then eluded in nuclease-free water for two independent pharmacogenomics assays. gDNA levels were quantified using a Qubit™ 4 Fluorometer (Invitrogen™, Cat No Q33238, Carlsbad, CA, USA) with the Qubit™ dsDNA HS Assay kit (Invitrogen™, Cat No 32854). DNA samples were stored at +4 °C for short-term (less than one month) or at −20 °C for long-term studies.

The HCL is accredited with the national GPRD (General Data Protection Regulation), protecting patients’ personal data. Furthermore, our laboratory adheres to the stringent requirements of the ISO 15189 accreditation (https://www.iso.org/fr/standard/76677.html), which we have received from the competent national authorities, including ARS (Agence Régionale de Santé) and COFRAC (Comité Français d’Accréditation). This accreditation confirms our commitment to upholding the highest standards of quality in medical laboratory practices, including the handling of personal health information and patient samples.

### 2.2. Customized Panel Combined with Illumina^®^ Short-Read Sequencing (SRS)

For validated SRS library preparation (SOPHiA GENETICS technique, Lausanne, Switzerland), 200 ng gDNA was used (capture technology from SOPHiA GENETICS), according to the manufacturer’s instructions [20]. The comprehensive panel covers 77 genes in routine use. The density of the probes was increased to discriminate *CYP2D6* and *CYP2D7*. The libraries were sequenced on NextSeq550 (Illumina, San Diego, CA, USA) in 2 × 150 paired-end runs. The bioinformatics analysis was performed using the SOPHiA DDM™ platform (SOPHiA GENETICS, Lausanne, Switzerland) [21]. *CYP2D6* bioinformatics analysis included customized phasing and haplotyping, aiming to differentiate between pseudogenes, hybrids, and different haplotypes. A bioinformatics analysis had been performed separately in-house for the other genes using the Stargazer bioinformatics tool [22].

The short-read sequencing (SRS) process is accredited under ISO 15189 by COFRAC (Comité Français d’Accréditation), which is a testament to the stringent quality standards we adhere to. The ISO 15189 accreditation requires rigorous and regular internal and external quality controls to be in place. These controls ensure that every aspect of the laboratory’s operations, including the sequencing process, meets the highest international standards for medical laboratories. In practical terms, this accreditation involves the continuous monitoring of our SRS processes through both internal quality checks and participation in external quality assessment schemes. These assessments are designed to objectively evaluate our performance and verify the accuracy of our sequencing results. By successfully maintaining this ISO 15189 accreditation, our laboratory demonstrates ongoing compliance with defined quality criteria and the competence to produce valid and reliable sequencing data. The accreditation process evaluates the entire workflow, including sample handling, data analysis, and reporting, thus providing a comprehensive validation of our sequencing services.

### 2.3. Twist Panel Combined with the PacBio SMRT^®^ Long-Read Sequencing (LRS)

The GENTYANE’s platform (INRAE, Clermont-Ferrand, France) performed a LRS assay with a limited gene panel, developed by Twist Bioscience. Probes were optimized using a proprietary algorithm to enable balanced capture of complex regions. Probes were designed to cover a 2 Mbp target region of interest with sparse tiling density at 0.1×. Among the covered genes, we focused on the *CYP2D6, UGT1A1,* and *NAT2* genes.

The impact of short-term storage at +4 °C on DNA integrity was carefully monitored by assessing the quality of the DNA at multiple time points in our study. We have conducted comprehensive DNA quality assessments using a Femto Pulse system from Algilent, an automated pulsed-field capillary electrophoresis instrument designed specifically to evaluate the integrity of high-molecular-weight DNA. The results from the Femto Pulse analysis confirm that the DNA samples, even after short-term storage at +4 °C, maintained high integrity, with no significant fragmentation or degradation observed.

For the Twist LRS library preparation, 300 ng gDNA was used (Long-read Library Preparation and Standard Hyb v2 Enrichment from Twist Bioscence, South San Francisco, CA, USA), according to the manufacturer’s instructions [23]. The long-read PGx Twist Panel covered 49 pharmacogenes involved in drug metabolism and therapeutic responses, and was designated and validated by academic collaborators [24]. The cumulative target length of the panel was 2 Mbp. The library preparation workflow includes mechanical fragmentation (three steps) and hybridization target enrichment (four steps). The gDNA fragmentation was performed with Megaruptor 3 (Diagenode SA, Ougrée, Belgium). Size distribution was performed with the Femto Pulse system with the Genomic DNA 165 kb kit (Agilent Technologies, Paris, France); each size control was performed on the Femto Pulse. End repair, the dA-Tailing, and adapter ligation were performed according to the manufacturer’s protocol [23]. Each adapter-ligated sample was purified using freshly prepared 80% ethanol, followed by size selection using DNA purification beads and elution into 37 μL of 10 mM Tris-HCl pH 8. Next, pre-capture amplification was performed using KOD Xtreme Hot Start DNA Polymerase (Merck, Darmstadt, Germany) in 154 μL total reaction volume containing 10 μL of molecular-biology-grade water, 100 μL of 2× Xtreme Buffer, and 40μL of dNTPs (2 mM each). The PCR parameters were 2 min at 94 °C, followed by eight cycles of 10 s at 98 °C, 30 s at 58.8 °C, and 10 min at 68 °C, with a final extension of 10 min at 68 °C. After amplification, each sample was purified using DNA purification beads followed by freshly prepared 80% ethanol and elution into 22 μL water, 10 mM Tris-HCl pH 8. The DNA concentration and size distribution of the amplified sample was assessed using Qubit Fluorometer dsDNA High Sensitivity Assay and the 2100 BioAnalyzer System using the Genomic DNA 12,000 kit, respectively. After quality control, samples were pooled in equimolar for a total mass per pool of 1500 ng (187.5 ng per library), with a maximum of eight samples in each pool. After pooling, samples were captured using Twist Hybridization, Twist Wash Kit, and Twist Long-read PGx panel according to the manufacturer’s instructions [23]. The captured pools (50 μL/sample) were amplified using KOD Xtreme Hot Start DNA Polymerase (Merck, Darmstadt, Germany) in 200 μL total reaction volume containing 6 μL of amplification primers, 100 μL of 2 × Xtreme Buffer, and 40 μL of dNTPs (2 mM each). The PCR parameters were 2 min at 94 °C, followed by 13 cycles of 10 s at 98 °C, 30 s at 58.8 °C, and 10 min at 68 °C, with a final extension of 10 min at 68 °C. After amplification, each sample was purified using DNA purification beads, followed by freshly prepared 80% ethanol and elution into 42 μL water, and 10 mM Tris-HCl pH 8. The DNA concentration and size distribution of the amplified sample was assessed using the Qubit Fluorometer dsDNA High Sensitivity Assay and the 2100 BioAnalyzer System, using the Genomic DNA 12,000 kit, respectively. The PacBio library was generated from the Twist capture product using the SMRTbell prep kit 3.0.

The SMRTbell libraries were sequenced on a Sequel II instrument (GENTYANE platform). The SMRTbell library was loaded at an on-plate concentration of 85 pM. The sequencing primer was annealed to the template at a molar ratio of 20:1 (primer:template) for 30 min at 20 °C. Circular Consensus Sequencing reads (“CCS reads”) were generated using CCS software version 3.0.0 (https://github.com/pacificbiosciences/unanimity/ (13 July 2022)) with “—minPasses 3—minPredictedAccuracy 0.99—maxLength 21,000”. Reads were mapped to the hg19/GRCh37 human reference genome. CCS reads were mapped using pbmm2 software version 0.10.0 (https://github.com/PacificBiosciences/pbmm2 (13 July 2022)) with “—preset CCS”. CLR reads were mapped using “pbmm2—preset SUBREAD”. NGS reads were mapped with minimap235 version 2.14-r883 with “-x sr”. To measure coverage by local [GC] content, bedtools49 version 2.27.1 was used to divide the hg19/GRCh37 reference genome into 500 bp windows “bedtools makewindows -w 500” and then to calculate the [GC] content “bedtools nuc” and average coverage “bedtools coverage -mean” of each window. The Dazzler suite (https://dazzlerblog.wordpress.com/ (13 July 2022)) was used to evaluate the accuracy of the CCS reads without relying on a reference genome. Briefly, “daligner27 commit 381fa920” was used to align pairs of CCS reads and produce all local alignments longer than 1 kb with less than 5% difference in sequence. Each CCS subject read was partitioned into 100 bp panels, within which its coverage by and concordance to other reads aligning to it was calculated. Panels with a concordance in the worst 0.1% of all panels were considered low quality. Abrupt ends in the alignment of five or more reads to a given panel along the CCS subject read were used to estimate library artifacts like chimeric molecules and missing adapters. A genome position was considered mappable if it was covered by alignments for at least ten reads at a specified mapping quality or higher, which was evaluated using “bedtools bamtobed” and “bedtools genomecov -bga”. Gaps (“N” base pairs in the reference) were excluded. Small variant calls were phased using WhatsHap v0.17 “WhatsHap phase”. The number of switch and Hamming errors were computed against trio-phased variant calls from GIAB using “WhatsHap compare”. To model the phase blocks achievable with a given read length, cuts were introduced between heterozygous variants in the GIAB trio-phased variant call set that were separated by more than the read length, which effectively assumes that adjacent heterozygous variants separated by less than the read length can be phased. Structural variant detection was done with pbsv version 2.1.0 (https://github.com/PacificBiosciences/pbsv (13 July 2022)) on pbmm2 CCS read alignments. The pbsv discover stage was run separately per chromosome with tandem repeat annotations (https://github.com/PacificBiosciences/pbsv/tree/master/annotations (13 July 2023)) “-tandem-repeats”. The pbsv call stage was run on the full genome.

## 3. Results

### 3.1. Global Genotype and Phenotype Concordances

**Figure 1:**
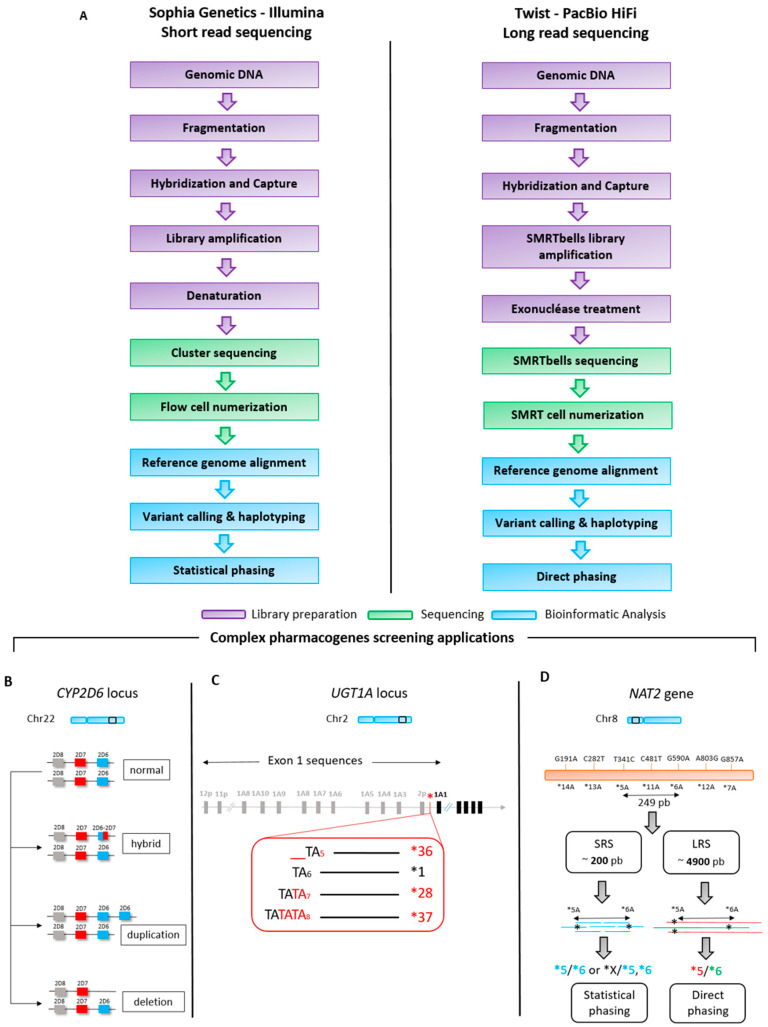
Short-read sequencing and long-read sequencing workflows (**A**) and schematic gene structure organization of *CYP2D6* (**B**)*, UGT1A1* (**C**), and *NAT2* (**D**). B. *CYP2D6* hybrid is a chimeric gene product resulting from the unequal crossover between two distinct *CYP2D6* and *CYP2D7* alleles. *CYP2D6* duplication refers to the presence of an extra copy of the *CYP2D6* gene in one or two parental alleles. CYP2D6 deletion denotes the absence of a copy of the *CYP2D6* gene in the genome. C. The UGT1A1*28 (TA7), *36 (TA5), and *37 (TA8) haplotypes are characterized by specific genetic variations in the UGT1A1 gene promoter, compared to the *1 haplotype which is considered to be the wild-type or reference allele. D. SRS: short-read sequencing, LRS: long-read sequencing. For the *NAT2* gene, which has a direct relationship between haplotype and phenotype, the direct phasing approach after LRS provides continuous, high-resolution haplotype information, whereas statistical phasing with short-read data relies on population-based inference and can be less accurate for variants (e.g., *5 and *6) with molecular distances superior to >200 pb (SRS fragment length).

The study was designed as a proof of concept to evaluate NGS 3rd generation of sequencing in pharmacogenomics in routine settings. It aims to perform a comprehensive performance comparison between the SRS technology (Illumina) and LRS technology (PacBio) for the analysis of three genes: *CYP2D6, UGT1A1,* and *NAT2* (Figure 1). The selection of these genes was based on their unique molecular characteristics that pose challenges for conventional sequencing methods. Specifically, the *CYP2D6* gene was chosen due to its high level of polymorphisms, which makes it a complex target for accurate variant calling (Figure 1A). The *UGT1A1* gene was included because it has a repeat sequence located in its promoter region, complicating the determination of TA repeat numbers (Figure 1B). Finally, the *NAT2* gene was selected for its specific genetic nomenclature that directly links genotype to phenotype, necessitating the phasing of parental alleles for accurate haplotype determination in the offspring (Figure 1C).

Thirteen samples were analyzed in SRS and LRS to determine the concordance of three parameters: haplotype, diplotype, and metabolism scoring (phenotype) (Table 1). A 38.4% concordance was found for haplotyping determination, 61.5% was found for diplotype determination, and 100% for metabolic score (AS) phenotype determination. We reported all haplotypes (*CYP1A2, CYP2B6, CYP2C19, CYP2C9, CYP2D6, CYP3A4, CYP3A5, TPMT, UGT1A1, UGT2B15,* and *NAT2)* from Table S2. One mismatch could be attributed to mis-haplotyping with the SRS methodology for *CYP2D6*, and four additional mismatches to a better phasing with LRS for *NAT2* analysis. Three other mismatches could be attributed to the low coverage/depth after LRS for *UGT1A1.* The main coverage deficiency was noted in two genomic regions pertaining to the UGT1A1 gene, specifically at positions UGT1A1*60 and UGT1A1*93. Both these positions exhibit linkage disequilibrium with the presence of haplotype UGT1A1*28, indeed located on the promoter of the UGT1A1 gene. In the three patients where a lack of coverage/depth was observed, the presence of the predominant UGT1A1*28 haplotype over UGT1A1*60 and UGT1A1*80 did not influence the predicted metabolism score of UGT1A1. With the current state of the art, while the coverage deficiencies at these specific loci are noteworthy, they did not adversely affect the prediction of metabolism score for these individuals. This lower coverage/depth issue affected only three cases and was isolated in two distal promoter genomic positions of UGT1A1.

### 3.2. CYP2D6 Analysis

**Figure 2:**
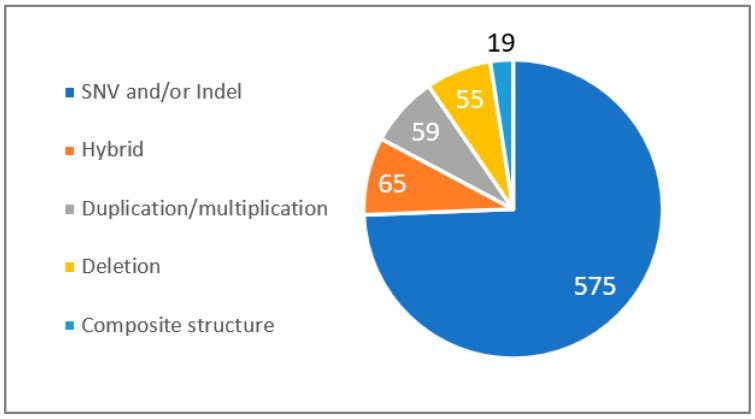
Prevalence of *CYP2D6* structural forms from the HCL hospital. SNV: single nucleotide variant, InDel: small insertions and small deletions. Composite structure: a composite structure of the CYP2D6 gene refers to a complex genetic arrangement, formed either from the coupling of a hybrid with a gene duplication, or the union of a hybrid with a gene deletion.

As is well known, the *CYP2D6* gene is highly polymorphic and heterogeneous in the general population. Among the HCL’s recruitment of 773 patients assessed for no clinical response or ADRs, 198 (25.6%) presented a complex form of *CYP2D6* genomic structure associated with the prediction of a modified enzyme function (Figure 2). Among these complex structural forms, we observed 65 hybrid cases (32.8%), 59 duplication/multiplication cases (27.7%), 55 deletion cases (28.1%), and 19 composite structure cases (9.6%).

Regarding the distribution of diplotypes for *CYP2D6* hybrids, fifty-four (83.1%) patients presented a CYP2D6-CYP2D7 hybrid *68, three patients presented a CYP2D6-CYP2D7 hybrid *36 (4.6%), four patients presented a CYP2D6-CYP2D7 hybrid *4N, one patient presented a CYP2D6-CYP2D7 hybrid *61 (1.5%), and two patients presented a CYP2D7-CYP2D6 hybrid *13 (3.1%) (Table 2). Concerning CYP2D6 duplications, fifty-six (94.9%) patients presented a duplication (N = 2) and three (5.1%) patients presented a multiplication (N = 3). 

Concerning patients with a complete deletion of allele, thirty-four (61.8%) patients presented a *5 haplotype combined with a normal clinical function haplotype (*1,*2,*35,*34), twenty (36.4%) patients presented a *5 haplotype combined with a decreased (*41,*17,*9,*10,*29) or no (*4,*3) clinical function haplotype, and one (1.8%) patient presented a *5 haplotype combined with an unknown (*28) clinical function haplotype. We identified cases where CYP2D6 composite structures were associated with duplication/deletion with a hybrid primarily involving haplotype *68 or hybrid duplication (e.g., *4 × 2,*68 × 2). For the “composite structure” category, the most prevalent form was the haplotype *4,*68 combined with duplication. A total of six patients were identified to have at least one supernumerary copy of this specific hybrid (*1/*4,*68 × 2,*4 × 2,*68 × 2,*1/*4,*68 × 3,*4/*28,*68 × 2). The CYP2D6 haplotyping was performed using the dedicated tool developed by SophiaGenetics. CNVs were confirmed with long-read PCR and MLPA techniques.

Among the thirteen selected samples analyzed with both techniques, six samples (Sample 10, Sample 3, Sample 11, Sample 1, Sample 4, and Sample 13) with atypical genetic profiles were selected based on CNV and structural forms (Table 3). A 92.3% (12/13) concordance was reported for diplotype determination and 100% (13/13) for phenotype determination based on the enzyme metabolic activity score (AS). For the Sample 4, the presence of a duplication of *CYP2D6* on the first allele (*2 × 2) was masked by the deletion of the CYP2D6 (*5) on the second allele. This deletion was confirmed by a classical targeted method (multiplex ligation-dependent probe amplification, MLPA) and an in-house semi-long PCR assay. For the five other samples, SRS and LRS findings were concordant for genotyping (haplotype and diplotype) and AS determination.

Concerning the SNV and/or indel group, a 100% (7/7) concordance was reported for diplotype and AS determination. For these samples presenting a simple form of *CYP2D6*, SRS and LRS findings were concordant.

### 3.3. UGT1A1 Analysis

The thirteen samples were analyzed by SRS and LRS on the TA repeated region at the g.23466879-23466881 genomic position on the Hg19/GRCh37 assembly. The *28 variant corresponds to seven TA repeats. Other alleles are also described at this location with different numbers of repeats: *1 (six TA repeats), *36 (five TA repeats), and *37 (eight TA repeats). Concerning diplotype determination and metabolizer status, a full concordance was found (Table 4). For three samples, Sample 2, Sample 5, and Sample 12, discrepancies were observed for the determination of haplotypes *60 and *93 between the SRS and LRS methods. For *60 and *93 haplotypes, homozygous status wild-type (Sample 2, Sample 5) was found in LRS while the SRS method found heterozygote status. Concerning the Sample 12, the comparison showed an absence of coverage for *60 and *93 positions. For these three patients, discordances were certainly due to the low sequencing depth using the LRS method (Sample 2: 2157× in SRS and 3× in LRS for *60 and 1501× in SRS and 3× in LRS for *93; Sample 5:1763× in SRS and 13× in LRS for *60 and :1271× in SRS and 13× in LRS for *93; Sample 12:3492× in SRS and 0× in LRS for *60 and :2725× in SRS and 0× in LRS for *93).

### 3.4. NAT2 Analysis 

**Figure 3:**
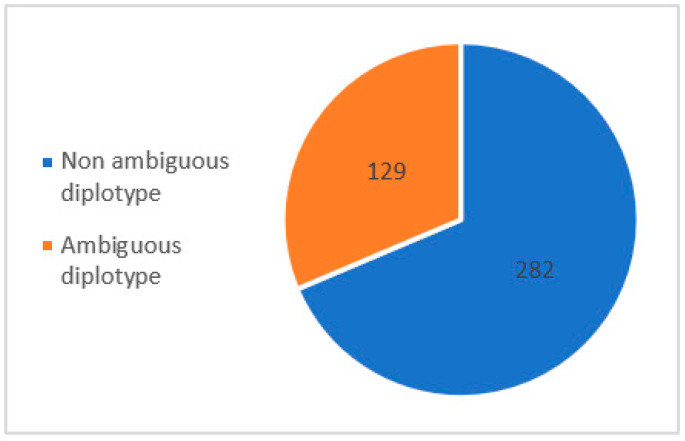
Prevalence of *NAT2* diplotypes from HCL’s hospital.

To compare the performance of direct phasing (LRS, WhatsHap) with statistical phasing (SRS, Stargazer), *NAT2*, a gene with a specific nomenclature depending on the location of each parental variant for phenotype determination, was chosen (Supplementary Table S1). *NAT2* is a 9936 bp gene composed of two exons of which only one is coding, including all single nucleotide variants (SNVs) of clinical interest, which allows for the production of a functional enzyme NAT2. These SNVs are reported according to the international nomenclature as a star allele haplotype, with NAT2*4 being a wild-type allele without the presence of any clinical SNV. Phasing consists of the precise *cis-* and *trans-* allele location determination. Due to the difficulty in obtaining a reliable genotype–phenotype prediction using classical experimental methods of molecular haplotyping, phasing by statistical inference is widely used to determine the location of parental haplotypes after SRS. However, this method may fail to give the exact diplotype for some ambiguous genetic profiles. Among a recruitment of 411 patients from the Hospices Civils de Lyon addressed for ADRs concerning NAT2 drug substrates, we found 282 (68.6%) non-ambiguous diplotypes and 129 (31.4%) ambiguous diplotypes with a putative impact on the metabolic scoring of *NAT2* (Figure 3, according to data included in the Supplementary Table S1).

A non-ambiguous diplotype is defined as a diplotype for which statistical phasing performed after SRS was able to attribute each haplotype identified on the parental alleles. The *cis-* and *trans-* determination is crucial for determining the acetylation metabolism score. This first category includes diplotypes comprising haplotypes linked to the rapid acetylator profiles (NAT2*4, NAT2*12, and NAT*13) and the slow acetylator profiles (NAT*5, NAT2*6, NAT2*7, and NAT2*14) found. These can be in various states: (i) homozygous wild-type state (e.g., *4/*4) or containing a rapid variant (e.g., *4/*12); (ii) homozygous variant state (e.g., *5,* 11,*12/*5,*11,*12); (iii) heterozygous state containing only one slow variant (e.g., *4/*5,*11,*12); (iv) composite heterozygote state containing two different slow variants with a physically allelic distance inferior to 200 bp (e.g., * 5,*12/*14) (Table 5).

An ambiguous diplotype is defined as one for which statistical phasing performed after SRS was not able to assign each identified haplotype on the parental alleles. This second category includes the diplotypes comprising the haplotypes linked to slow acetylator profiles (NAT*5, NAT2*6, NAT*7, and NAT*14) found. These may be in various states: (i) heterozygote state with two slow variants phased on the same parental allele (e.g., *5,*6,*11/*12,*13), or (ii) composite heterozygote state (e.g., *5,*11,*12/*6,*13). The objective of this part was to compare the two phasing techniques specific to each of the sequencing methods used (SRS—statistical phasing and LRS—direct phasing) for the determination of the clinical diplotypes of our thirteen patients for the *NAT2* gene. Here, *NAT2* clinical diplotypes were determined for our thirteen samples, to compare methods between SRS and LRS (Table 6). Among these patients, twelve presented with a non-ambiguous diplotype and one had an ambiguous diplotype.

**Figure 4:**
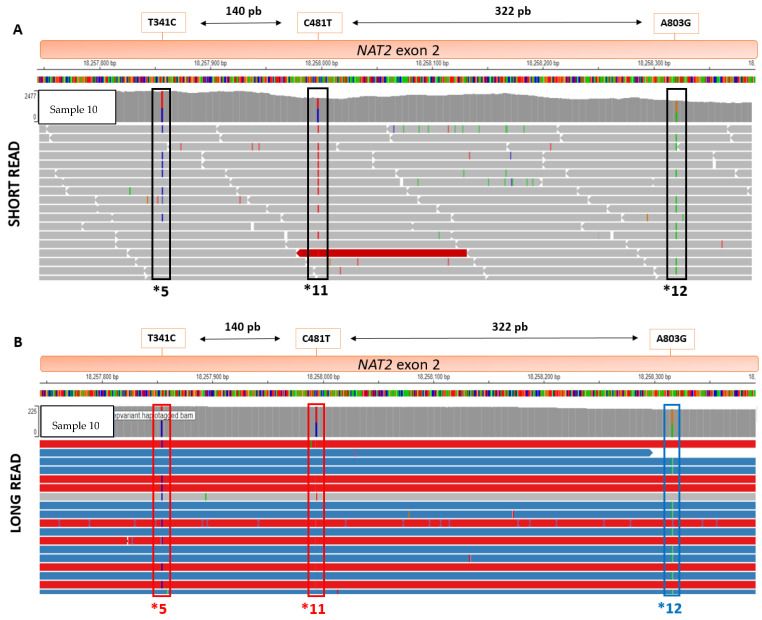
Comparison of fragment length, in SRS (**top**) and LRS (**bottom**), for *NAT2* haplotypes *5, *11, and *12: direct phasing with WhatsHap module allows parental haplotype identification (red and blue fragments) using Integrative Genome Viewer (IGV). (**A**) Haplotypes *5,*11 and *12 viewed on SRS BAM using IGV. (**B**) Haplotypes *5,*11 and *12 viewed on LRS BAM using IGV.

Using data from the analysis after LRS using the WhatsHap software, read-based phasing enabled confirmation of the concordance of the *NAT2* diplotypes, as well as the resulting NAT2 acetylator status. For four non-ambiguous samples and one ambiguous sample, clinical diplotype discordances were highlighted. For the Sample 3 and Sample 11, direct phasing analysis showed that the haplotypes *6 and *13 were all located on the same parental allele. For Sample 9 and Sample 4, direct phasing analysis showed that the haplotypes *5 and *11 were all located on the same parental allele. For Sample 10, the location of *12 was corrected on the other parental allele after direct phasing (Figure 4).

Despite a clinical diplotype change of the ambiguous sample, the phenotype remained unchanged.

## 4. Discussion

Drug safety and efficacy is of critical importance in various medical fields, particularly psychiatry, oncology, and infectious diseases. Local variations in pharmacogenetics could result in a high proportion of patients receiving ineffective treatments or having adverse effects. Moreover, variations in pharmacogenomics may place an unacceptably high proportion of individuals at risk of serious complications from treatment. In this context, pre-therapeutic pharmacogenetic analysis could significantly reduce the appearance of adverse effects in patients treated with drugs with a narrow therapeutic range [25,26]. To assess our LRS method, we analyzed atypical samples from patients. In this study, we conducted a retrospective comparison between PacBio LRS using a Twist Bioscience hybrid capture panel and Sophia Genetics-Illumina SRS using a customized in-house panel of eleven pharmacogenes (Supplementary Table S2), including complex genes *CYP2D6*, *UGT1A1,* and *NAT2* with known genotypes. For most of these, we successfully identified SNVs, CNVs, structural variations, and phased haplotypes. For these genes, LRS was compared to SRS, which previously provided consensus diplotypes including star (*) allele haplotypes and metabolizer status on clinical samples. In addition to predicting metabolizer status for *CYP2D6*, *UGT1A1,* and acetylator status for *NAT2*, direct phasing with LRS highlighted samples with discrepant or unclear haplotype configurations from statistical phasing after SRS. This suggests that LRS should have significant utility for clinical testing. Although targeted genotyping of these genes in clinical settings is widely available using SRS platforms, clinical diplotype determination remains challenging, due to the presence of complex genomic regions for genes such as *CYP2D6*, *UGT1A1,* and *NAT2.* First, we analyzed the cytochrome P450 2D6 (*CYP2D6*), which is a phase I enzyme and a subfamily member of the CYP450 superfamily involved in the oxidative metabolism of endogen compounds and xenobiotics [27]. Quantitatively, *CYP2D6* represents approximatively 1% to 4% of all hepatic CYP450 enzymes and metabolizes approximately 25% of medicines authorized for prescription by international agencies (e.g., anti-cancer agents, anti-depressants, anti-psychotics, and opioids) [27]. For *CYP2D6* analysis, combining the dedicated bioinformatics pipeline with copy number analysis, we identified one sample which is likely to be *2 × 2/*5 in LRS instead of *2/*2 in SRS. This sample presented an association with a duplicated wild-type functional haplotype, and the haplotyping short-read software (Stargazer, https://doi.org/10.1002/cpt.1552, version 1) failed to detect the *5 haplotype. 

Stargazer is primarily designed to deduce star alleles based on single nucleotide variants and small indels by comparing read patterns to reference haplotypes. The tool’s algorithm, however, is not optimally configured in our pipeline to interpret the complex genomic rearrangements that characterize these two haplotypes simultaneously. The presence of a gene duplication can mask the signal of a deletion, due to the additional copies of the gene, leading to a misinterpretation of the allelic composition. This represents a known challenge in pharmacogenomic analysis, as the accurate detection of such structural variations requires a level of resolution that can differentiate between multiple gene copies and the absence thereof, which is beyond the current capability of Stargazer.

To ensure the accuracy of our findings, each haplotype was not solely reliant on the initial computational predictions. We conducted a manually cured visualization of BAM files. This step allowed us to manually review and confirm the presence of structural variants and their respective haplotypes as indicated by the sequencing reads themselves. By directly examining the aligned sequencing data, we were able to cross-validate the variant calls, providing an additional layer of assurance in our results. This manual verification process is crucial, especially when dealing with complex pharmacogenes, as it allows for the identification and correction of potential mis-calls that computational tools may generate. 

However, as discussed previously, this error had in our cases no impact on the AS for the patients but had an impact for the children. In the context of our study, which also aimed to identify the prevalence of complex forms of the *CYP2D6* gene in a hospital-based population, several factors warrant careful consideration, especially when compared to the general population. First, the role of ethnic background cannot be overstated. For instance, *CYP2D6* gene duplications are notably more frequent in African-American and Ashkenazi Jewish populations [27,28], which can considerably affect the observed prevalence depending on the ethnicity of the sample. Second, it is crucial to acknowledge the inherent recruitment bias present in a hospital setting. The patients we encounter are more likely to experience therapeutic ineffectiveness or adverse effects, particularly those on psychotropic medications that are metabolized by CYP2D6. This can artificially inflate the prevalence of complex *CYP2D6* forms compared to the general population, where fewer people may be experiencing these specific health challenges. 

The uridine diphosphate glucuronosyltransferase 1A1 (*UGT1A1*) is a phase II enzyme and a member of the UDP-glucuronosyltransferases (UGTs) subfamily involved in the conjugation of endogenous compounds and xenobiotics. In clinical practice, *UGT1A1* assumes an important role in drug metabolism, due to the glucuronidation of bilirubin. This transferase is the rate-limiting step in ensuring efficient bilirubin clearance, and this rate can be affected by both genetic variations and competing substrates (e.g., irinotecan) [8,9]. Patients receiving irinotecan therapy are predisposed to hematological and gastrointestinal toxicities resulting from elevated serum levels of the active irinotecan metabolite (SN-38). Thus, the net result of insufficient UGT1A activity is the subsequent accumulation of a toxic drug in the GI epithelium. For TA repeated sequence analysis, we showed a total concordance for all patients presenting with a TA repeated variant (UGT1A1*28, UGT1A1*37) responsible for an AS decrease, resulting in an increased risk of severe toxicity after irinotecan administration. For this gene, it is important to differentiate between variants that exert a functional impact, and those that are commonly associated with other polymorphisms. In particular, the *60 variant is known to have a functional impact on the enzymatic activity of UGT1A1, affecting its role in drug metabolism and bilirubin conjugation. On the other hand, variants like *80 and *93 are often found in association with *28 but do not independently contribute to an altered enzyme function. Therefore, when studying the functional implications of *UGT1A1* polymorphisms, it is crucial to focus on those variants that directly alter the gene’s functionality.

For *NAT2* clinical diplotype determination, SRS requires a statistical phasing tool (Stargazer^®^) to determine *cis-* and *trans-* haplotype configurations [11,29]. For *NAT2* analysis, we compared SRS statistical phasing with LRS direct phasing for patient haplotype determination. After phase processing (WhatHap^®^), we were able to correct, for four patients, the initial SRS haplotyping using the free access Integration Genome Viewer (IGV) tool. More precisely, the distance separating each of the haplotypes present in the patients each time exceeded the size (>200 pb) of the SRS fragments (308 bp for *13−*6 distance, 462 bp for *12−*5 distance, 322 bp for *12−*11 distance, 500 bp for *11−*5 distance). This fact explains why clinical haplotypes could be corrected after direct phasing of the fragments obtained in LRS (length~4985 bp), underlining the advantage of the LRS approach compared to SRS. Using different sequencing platforms can offer specific advantages for haplotype phasing, mainly for genes like *NAT2*, where variant phasing directly influences enzymatic activity and metabolic capacity. For instance, the Illumina NEXTseq 500 typically generates read lengths of up to 200 bp, but the Illumina MiSeq instrument can sequence longer reads, up to 300 bp, but for a small number of patients per run. These longer fragments provide benefits close to those from the LRS, which allow the capture of more variants within a single read, thus enhancing our ability to phase variants accurately, and determine the functional implications for the NAT2 enzyme. LRS technology offers a robust and expedient approach to haplotyping that simplifies the complexities often associated with both intricate and simple genes that may have numerous variant imbalances. Its ability to read extended stretches of DNA in a single run enables the capture of multiple variants together, thereby facilitating direct phasing. This eliminates much of the uncertainty and computational complexity often required for short-read methods, especially for genes with intricate structural variations or for those where multiple variants are in nearby genomic locations. Therefore, LRS provides a streamlined, unambiguous method for haplotyping, making it a particularly valuable tool in genetic studies where quick and reliable results are paramount.

Our study demonstrated that, despite notable differences in haplotype and diplotype determination between SRS and LRS, no significant discrepancies were observed in phenotype prediction for the analyzed patient samples. This outcome is particularly reassuring given that the samples were derived from patients who had been prescribed pharmacogenetic analysis. It suggests that the current SRS method is robust, even though it may exhibit certain limitations in the precise determination of some haplotypes. The key takeaway from our findings is that LRS facilitates more accurate haplotype detection and does so with a lower demand for bioinformatic resources. This efficiency is largely attributable to the capacity of LRS to determine variant locations directly from BAM files without extensive bioinformatics processing. The phasing software, WhatsHap, is instrumental in this aspect as it generates BAM files annotated with parental haplotypes, enhancing our ability to pinpoint haplotype locations with greater precision. Although in our cohort, the focus on complex haplotypes was confined to genes *CYP2D6*, *UGT1A1*, and *NAT2*, the precise localization of clinical variants in other pharmacokinetic genes through LRS can enable the determination of more accurate haplotypes and diplotypes with minimal bioinformatic resources. Lastly, it is important to consider that inaccuracies in haplotype determination could have implications for the prediction of a metabolism score in the offspring of individuals. Inaccurate haplotype predictions could potentially affect future pharmacogenetic implications for the descendants. In conclusion, while both SRS and LRS provide equivalent results at the phenotype level, for the patients in our study, LRS offers a finer resolution in haplotyping which can be important for comprehensive pharmacogenetic analysis, particularly in complex genes and in predicting genetic inheritance patterns. Our study thus underscores the potential of LRS for enhancing the precision of pharmacogenomic applications while acknowledging the currently effective implementation of SRS in clinical settings.

Despite an unchanged prediction concerning the AS, the LRS approach allowed us to correct complex *CYP2D6* and *NAT2* clinical diplotypes. The comparison between LRS and SRS strategies highlights the advantage of direct phasing in the accurate and in-depth understanding of pharmacogenomics analysis. While SRS is still widely used for its robustness and analytical accuracy in medical settings, LRS could offer a more comprehensive approach by allowing the reconstruction of complex genomic regions and providing more detailed information on genetic structure and variations [30]. One of the main advantages of the LRS strategy is its ability to generate longer sequence reads, often several thousand base pairs. This feature overcomes the limitations of short sequences, which can lead to editing errors and difficulty in resolving repeated regions or genomic regions with high similarity [31]. By obtaining longer reads, LRS facilitates more accurate and complete reconstruction of complex genomic regions. Another major advantage of LRS could be its ability to provide direct allele phasing for certain pharmacogenes analyzed in medical practice. Unlike SRS, which requires additional effort to resolve phasing using statistical phasing or population phasing approaches, LRS allows for the direct and reliable determination of alleles [32]. This is especially beneficial for the accurate identification of genetic variations, SNVs, InDel, repeated sequences, CNVs, and structural variants. Furthermore, the direct phasing enabled by LRS could play crucial roles in areas such as precision genomics, the study of inherited diseases, and the identification of genetic markers. It could enable a deeper analysis of haplotypes and associated phenotypes, which is essential for understanding complex genetic mechanisms and developing personalized diagnostic and treatment strategies. 

Here, we provide a thorough comparison of SRS and LRS within the realm of clinical testing, considering key aspects that influence their application in a healthcare setting. Cost: Traditionally, SRS has been the more cost-effective option for routine clinical applications due to its established workflows and economies of scale. However, LRS costs have been decreasing and are expected to become more competitive as the technology matures and is adopted more widely. The precise cost-effectiveness of LRS is also influenced by its higher accuracy in complex genomic regions, which can potentially reduce the need for supplementary testing. Turn-around time (TAT): SRS benefits from shorter TATs, largely because it is well-integrated into clinical laboratory workflows with established infrastructures. LRS, while typically associated with longer TATs due to its nascent integration into clinical settings, offers the potential for reduced overall analysis time. This is attributed to its ability to resolve complex genomic regions without the need for additional confirmatory testing steps, such as Sanger sequencing for complex variants. Nevertheless, in France, clinical genetics platforms are moving towards LRS workflows, to facilitate the identification of structural forms. Accuracy and precision: As highlighted in our study, LRS demonstrates superior precision in haplotyping, particularly for complex genes such as *CYP2D6*, *UGT1A1*, and *NAT2*. While SRS is robust and reliable for phenotype predictions, LRS offers an enhanced resolution that can be critical for comprehensive pharmacogenetic analyses and for future applications where precise gene modeling is required. Bioinformatic resources: LRS can reduce the need for extensive bioinformatics support, due to its simplified data analysis pipeline. The direct determination of variant locations from BAM files using software such as WhatsHap simplifies the phasing process. This could translate into lower long-term costs and less labor-intensive operations as compared to SRS, where complex bioinformatics pipelines are often necessary to resolve ambiguities in haplotype phasing. This requires greater data storage capabilities, although the cost of data storage is continually decreasing. Clinical impact: Both SRS and LRS can provide clinically actionable results. However, LRS’s higher accuracy in detecting structural variants and resolving complex haplotypes could improve clinical outcomes by enabling a more nuanced interpretation of pharmacogenomic data.

In summary, LRS, with its distinct advantage of direct phasing, could offer a new and powerful perspective in the field of genomics. By combining longer reads and precise phasing, this approach paves the way for significant advances in our ability to individually treat patients based on their pharmacogenetic profile, but also in our understanding of the field of genomics. Its increasing use in translational research promises to stimulate new discoveries and innovative clinical applications in the era of precision medicine.

Recently, to consolidate pharmacogenetics knowledge, these strategies allow prospective randomized-trial-linked genetics signatures to be related to functional impacts. It demonstrated that pharmacogenetic-orientated prescription improves the prediction of drug response, while dramatically decreasing the incidence of clinically relevant ADRs in large-scale implementation, to significantly improve drug therapy management [26].

## Figures and Tables

**Table 1:** Concordance between SRS (Stargazer) and LRS (WhatsHap) methods for three parameters: haplotype, diplotype, and phenotype. SRS: short-read sequencing, LRS: long-read sequencing. The genes reported are *CYP1A2, CYP2B6, CYP2C19, CYP2C9, CYP2D6, CYP3A4, CYP3A5, TPMT, UGT1A1, UGT2B15,* and *NAT2*.

Samples (n = 13)	Haplotype Concordance SRS vs. LRS	Diplotype Concordance SRS vs. LRS	Phenotype Concordance SRS vs. LRS	Details
Sample 1	yes	yes	yes	
Sample 2	no	yes	yes	Low depth for UGT1A1*60 and UGT1A1*93 by LRS (TA6/TA7)
Sample 3	no	no	yes	NAT2*6/*13 given by statistical phasing was detected as NAT2*4/*6,*13 by direct phasing
Sample 4	no	no	yes	CYP2D6*2/*2 by SRS was detected as *5/*2 × 2 by LRS NAT2 *5,*12,*13/*6,*11 given by statistical phasing was detected as NAT2 *6,*13,*12/*5,*11 by direct phasing
Sample 5	no	yes	yes	Low depth for UGT1A1*60 and UGT1A1*93 by LRS phasing (TA6/TA7)
Sample 6	yes	yes	yes	
Sample 7	yes	yes	yes	
Sample 8	yes	yes	yes	
Sample 9	no	no	yes	NAT2 *5,*12/*11 given by statistical phasing was detected as NAT2 *12/*5,*11 by direct phasing
Sample 10	no	no	yes	NAT2*4/*5,*11,*12 given by statistical phasing was detected as *12/*5,*11 by direct phasing
Sample 11	no	no	yes	NAT2*6/*13 given by statistical phasing was detected as NAT2 *12/*6,*13,*12 by direct phasing
Sample 12	no	yes	yes	No coverage for UGT1A1*60 and UGT1A1*93 with the LRS technique (TA6/TA7)
Sample 13	yes	yes	yes	
Total	38.4% (5/13)	61.5% (8/13)	100% (13/13)	

**Table 2:** Spread of diplotypes for *CYP2D6* complex forms.

CYP2D6 Structural Forms (n)	Diplotypes (n)
Hybrid (65)	*1/*4,*68 (16); *2/*4,*68 (14); *4/*41,*68 (7); *4 × 2,*68 (5); *1/*4,*4N (3); *4/*4,*68 (3); *3/*4,*68 (1); *1/*2,*68 (2); *9/*4,*68 (2); *4/*35,*68 (2); *1/*2,*13 (2); *41/*4,*68 (1); *2/*41,*68 (1); *4/*1,*61 (1); *4/*6,*4N (1); *10/*10,*36 (1); *4 × 2,*36 (1); *2/*10,*36 (1); *17/*45,*68 (1)
Duplication/multiplication (59)	*1/*2 × 2 (20); *2 × 2/*41 (6); *2 × 2/*4 (4); *2/*2 × 2 (4); *1 × 3 (3); *1/*4 × 2 (2); *2 × 3 (2); *10 × 2/*1 (1); *1 × 2/*53 (1); *4/*28 × 2 (1); *2 × 2/*28 (1); *2 × 2/*10 (1); *41/*71 × 2 (1); *2 × 2/*35 (1); *4 × 2/*9 (1); *1/*2 × 3 (1); *1 × 2/*9 (1); *1 × 2/*29 (1); *1 × 2/*41 (1); *2 × 3/*29 (1); *35 × 3 (1); *4/*9 × 2 (1); *3 × 2/*41 (1); *2 × 2/*17 (1); *2 × 2/*33 (1)
Deletion (55)	*1/*5 (25); *4/*5 (7); *2/*5 (6); *5/*41 (4); *5/*35 (2); *5/*17 (2); *5/*9 (2); *3/*5 (2); *5/*10 (2); *5/*34 (1); *5/*29 (1); *5/*28 (1)
Composite structure (19)	*2 × 2/*4,*68 (5); *1/*4,*68 × 2 (2); *4 × 2,*68 × 2 (2); *1 × 2/*4,*68 (1); *5/*4,*68 (1); *1/*4,*68 × 3 (1); *3/*4,*68 (1); *4/*28,*68 × 2 (1); *2 × 3/*68 (1); *2/*5,*36 (1); *2 × 2/*41,*80 (1); *10 × 2,*36 × 2 (1); *4 × 2,*4N × 2 (1)

**Table 3:** Comparison between SRS and LRS techniques of CYP2D6 concerning structural variant CNV detection. SRS: short-read sequencing, LRS: long-read sequencing, AS: activity score, CNV: copy number variation, UM: ultra-rapid metabolizer, NM: normal metabolizer, IM: intermediate metabolizer, PM: poor metabolizer.

CYP2D6 Form (n)	Sample (n = 13)	Copy Number Variation 2D6/2D7 /Hybrid	Hybrid	Diplotype	Metabolizer Status (AS)	Diplotype Concordance	Phenotype Concordance
Hybrid (1)	Sample 10	2/2/1	CYP2D6-2D7	*4/*41,*68	PM (0.5)	yes	yes
Duplication (2)	Sample 3	3/2/0	absence	*2 × 2/*41	NM (2.25)	yes	yes
	Sample 11	3/2/0	absence	*1/*4 × 2	IM (1.0)	yes	yes
Deletion (1)	Sample 1	1/2/0	absence	*5/*17	PM (0.5)	yes	yes
Deletion and duplication (1)	Sample 4	3/2/0	absence	*2 × 2/*5	IM (1.0)	no	yes
Composite structure (1)	Sample 13	3/2/1	CYP2D6-2D7	*1 × 2/*4,*68	NM (2.0)	yes	yes
SNV and/orindel (7)	Sample 12	2/2/0	absence	*1/*41	NM (1.25)	yes	yes
	Sample 9	2/2/0	absence	*29/*43	IM (1.0)	yes	yes
	Sample 8	2/2/0	absence	*1/*10	NM (1.25)	yes	yes
	Sample 7	2/2/0	absence	*4/*6	PM (0)	yes	yes
	Sample 6	2/2/0	absence	*1/*41	NM (1.25)	yes	yes
	Sample 5	2/2/0	absence	*9/*41	PM (0.5)	yes	yes
	Sample 2	2/2/0	absence	*2/*2	NM (2.0)	yes	yes

**Table 4:** Comparison between SRS and LRS techniques for the TA repeat sequence in the *UGT1A1* promoter. SRS: short-read sequencing, LRS: long-read sequencing, AS: activity score, NM: normal metabolizer, IM: intermediate metabolizer, PM: poor metabolizer.

Sample (n = 13)	Diplotype SRS	Metabolizer Status (AS) SRS	TA Repeat rs34983651 rs57191451 SRS vs. LRS	Diplotype LRS	Metabolizer Status (AS) LRS	Diplotype Concordance SRS vs. LRS
Sample 1	*36/*60	NM(2.125)	TA5/TA6 vs. TA5/TA6	*36/*60	NM (2.125)	yes
Sample 2	*1/*28	IM(1.3)	TA6/TA7 vs. TA6/TA7	*1/*28	IM (1.3)	yes
Sample 3	*1/*60	IM(1.7)	TA6/TA6 vs. TA6/TA6	*1/*60	IM (1.7)	yes
Sample 4	*1/*1	NM(2)	TA6/TA6 vs. TA6/TA6	*1/*1	NM (2)	yes
Sample 5	*1/*28	IM(1.3)	TA6/TA7 vs. TA6/TA7	*1/*28	IM (1.3)	yes
Sample 6	*1/*1	NM(2)	TA6/TA6 vs. TA6/TA6	*1/*1	NM (2)	yes
Sample 7	*28/*28	PM(0.6)	TA7/TA7 vs. TA7/TA7	*28/*28	PM (0.6)	yes
Sample 8	*1/*28	IM(1.3)	TA6/TA7 vs. TA6/TA7	*1/*28	IM (1.3)	yes
Sample 9	*36/*37	IM(1.3)	TA5/TA8 vs. TA5/TA8	*36/*37	IM (1.3)	yes
Sample 10	*1/*28	IM(1.3)	TA6/TA7 vs. TA6/TA7	*1/*28	IM (1.3)	yes
Sample 11	*1/*28	IM(1.3)	TA6/TA7 vs. TA6/TA7	*1/*28	IM (1.3)	yes
Sample 12	*1/*28	IM(1.3)	TA6/TA7 vs. TA6/TA7	*1/*28	IM (1.3)	yes
Sample 13	*28/*28	PM(0.6)	TA7/TA7 vs. TA7/TA7	*28/*28	PM (0.6)	Yes

**Table 5:** Spread of *NAT2* clinical diplotypes.

Type (n)	Diplotypes (n)
Non-ambiguous diplotype (282)	*5,*11,*12/*5,*11,*12 (70); *4/*5,*11,*12 (53); *6,*13/*6,*13 (42); *4/*6,*13 (33); *4/*4 (20); *6/*13 (14); *5/*11,*12 (7); *4/*5,*12 (5); *5,*11/*12 (5); *4/*12 (4); *7/*13 (4); *5,*11,*12/*5,*12 (3); *4/*7,*13 (2); *5,*11/*5,*11,*12 (2); *5,*12/*11 (2); *5,*12/*11,*12 (2); *6,*12/*13 (2); *4/*14,*13 (1); *4/*5,*11 (1); *4/*6,*12,*13 (1); *4/*6 (1); *5,*11,*12/*11,*12 (1); *5,*11,*12/*13 (1); *5/*11,*12,*13 (1); *5/*12 (1); *6,*13/*13 (1); *5,*12/*14 (1); *5,*14,*11,*12/*13 (1); *5/*14,*11,*12,*13 (1)
Ambiguous diplotype (129)	*5,*11,*12/*6,*13 (68); *5,*11,*12,*13/*6 (7); *5,*11/*6,*12,*13 (7); *5,*11,*12/*7,*13 (6); *5,*6,*11/*12,*13 (6); *5,*11/*6,*13 (6); *4/*5,*6,*11,*12,*13 (4); *5,*12/*6,*13 (4); *5,*6,*13/*11,*12 (3); *5,*6,*11,*12/*13 (3); *5,*13/*6,*11,*12 (2); *4/*5,*14,*11,*12 (1); *4/*6,*14,*13 (1); *5,*11,*12,*13/*7 (1); *5,*12,*13/*6 (1); *5,*12,*13/*6,*11 (1); *5,*14,*12/*11 (1); *5,*6,*11/*13 (1); *5,*6,*12/*11,*13 (1); *5,*6,*12/*13 (1); *5/*6 (1); *5/*7,*11,*12,*13 (1); *6,*13/*7,*13 (1); *6/*14,*13 (1)

**Table 6:** Comparison between short-read and long-read methods for *NAT2* analysis concerning phasing. SRS: short-read sequencing, LRS: long-read sequencing, AS: activity score, R: rapid acetylator; I: intermediate acetylator; S: slow acetylator.

NAT2 Form (n)	Sample (n = 13)	Diplotype SRS (Stargazer)	Acetylator Status SRS	Clinical Diplotype LRS	Metabolizer Status (AS)	Diplotype Concordance SRS|LRS
Non-ambiguous diplotype (12)	Sample 1	*4/*5,*12	IA	*4/*5,*12	IA	yes
	Sample 2	*4/*4	RA	*4/*4	RA	yes
	Sample 3	*6/*13	IA	*4/*6,*13	IA	no
	Sample 5	*5,*11,*12/*5,*11,*12	SA	*5,*11,*12/*5,*11,*12	SA	yes
	Sample 6	*4/*5,*11,*12	IA	*4/*5,*11,*12	IA	yes
	Sample 7	*4/*6,*13	IA	*4/*6,*13	IA	yes
	Sample 8	*5,*11,*12/*5,*12	SA	*5,*11,*12/*5,*12	SA	yes
	Sample 9	*5,*12/*11	SA	*12/*5,*11	SA	no
	Sample 10	*4/*5,*11,*12	IA	*12/*5,*11	IA	no
	Sample 11	*6/*13	IA	*12/6,*13,*12	IA	no
	Sample 12	*5,*11,*12/*5,*11,*12	SA	*5,*11,*12/*5,*11,*12	SA	yes
	Sample 13	*6,*13/*6,*13	SA	*6,*13/*6,*13	SA	yes
Ambiguous diplotype (1)	Sample 4	*5,*12,*13/*6,*11	IA	*6,*13,*12/*5,*11	IA	no

## Data Availability

The data presented in this study are available in the Supplementary Materials and from the corresponding author.

## Supplementary Materials

The following supporting information can be downloaded at: https://www.mdpi.com/article/10.3390/jpm13121655/s1, Table S1: Distance (in base pairs between successive SNVs in exon 2 of the NAT2 gene. Table S2: Comparison between short-read and long-read methods for *CYP1A2, CYP2B6, CYP2C19, CYP2C9, CYP3A4, CYP3A5, DPYD, TPMT, UGT2B15*.
