# Correction: Barthélémy et al. Direct Comparative Analysis of a Pharmacogenomics Panel with PacBio Hifi^®^ Long-Read and Illumina Short-Read Sequencing. *J. Pers. Med.* **2023**, *13*, 1655

**DOI:** 10.3390/jpm14101028

**Published:** 2024-09-26

**Authors:** David Barthélémy, Elodie Belmonte, Laurie Di Pilla, Claire Bardel, Eve Duport, Veronique Gautier, Léa Payen

**Affiliations:** 1 Institut of Pharmaceutical and Biological Sciences of Lyon, Claude Bernard Lyon I, 69373 Lyon, France; david.barthelemy@chu-lyon.fr (D.B.); claire.bardel@chu-lyon.fr (C.B.); 2 Department of Biochemistry and Molecular Biology, Lyon-Sud Hospital, Hospices Civils de Lyon, Réseau Francophone de Pharmacogénétique (RNPGx), 69495 Pierre-Bénite, France; laurie.di-pilla@chu-lyon.fr (L.D.P.); eve.duport@chu-lyon.fr (E.D.); 3 Center for Innovation in Cancerology of Lyon (CICLY) EA 3738, Faculty of Medicine and Maieutic Lyon Sud, Claude Bernard University Lyon I, 69921 Oullins, France; 4 Plateforme Génotypage et Séquençage en Auvergne (GENTYANE) UMR 1095 Génétique, Diversité Ecophysiologie des Céréales INRAE, Université Clermont Auvergne, 63100 Clermont Ferrand, France; elodie.belmonte@inrae.fr (E.B.); veronique.gautier@inrae.fr (V.G.); 5 Department of Bioinformatics, Hospices Civils de Lyon, 69008 Lyon, France

In the original publication [1], an error occurred where insufficient anonymization of samples was provided. The samples’ identifiers have been corrected and anonymized using the identifiers “Sample 1–13” and updated accordingly in Sections 3.2–3.4. The same identifiers have also been updated in Table 1, Table 3, Table 4, Table 6, Figure 4, and Table S2 in the Supplementary Materials.

The authors state that the scientific conclusions are unaffected. These corrections were approved by the Academic Editor. The original publication and the Supplementary Materials have also been updated.
