# Supplementary material for: Direct Comparative Analysis of a Pharmacogenomics Panel with PacBio Hifi® Long-Read and Illumina Short-Read Sequencing
Source: J Pers Med. 2023 Nov 27;13(12):1655. doi: 10.3390/jpm13121655 (PMC10744512; doi:10.3390/jpm13121655)
Supplement: Supplementary file 1 [file jpm-13-01655-s001.zip › jpm-2698327-supplementary.pdf]

Supplementary Table S1: Distance (in base pairs) between successive SNVs in exon 2 of the NAT2 gene. SNVs: Single Nucleotide variants.

Genomic position (chr8)	rs number	c.DNA	AS	Star Allele	*19	*14	*13	*5	*17	*11	*6	*12	*18	*7
18257703	rs1805158	190C>T,A	TT = Slow	*19		-1	-92	-151	-244	-291	-400	-613	-655	-667
18257704	rs1801279	191G>A	AA = Slow	*14	1		-91	-150	-243	-290	-399	-612	-654	-666
18257795	rs1041983	282C>T	TT = Rapid	*13	92	91		-59	-152	-199	-308	-521	-563	-575
18257854	rs1801280	341T>C	CC = Slow	*5	151	150	59		-93	-140	-249	-462	-504	-516
18257947	rs72554616	434A>C	CC = Slow	*17	244	243	152	93		-47	-156	-369	-411	-423
18257994	rs1799929	481C>T	TT =Rapid	*11	291	290	199	140	47		-109	-322	-364	-376
18258103	rs1799930	590G>A	AA = Slow	*6	400	399	308	249	156	109		-231	-255	-267
18258316	rs1208	803A>G	GG =Rapid	*12	613	612	521	462	369	322	213		-42	-54
18258358	rs56054745	845A>C	CC =Rapid	*18	655	654	563	504	411	364	255	42		-12
18258370	rs1799931	857G>A	AA =Slow	*7	667	666	575	516	423	376	267	54	12	

Supplementary Table S2: Comparison between short-read and long-read methods for *CYP1A2*, *CYP2B6*, *CYP2C19*, *CYP2C9*, *CYP3A4*, *CYP3A5*, *DPYD*, *TPMT*, *UGT2B15*.

	CYP1A2<br>(7 757 bp)<br>SRS | LRS<br>*1F: chr15:75041917C>A<br>*1C: chr15:75038220:G>A<br>*1L: chr15:75039612:AT>A<br>*1L: chr15:75047426:T>C	CYP2B6<br>(27 170 bp)<br>SRS | LRS<br>*2:chr19:41497129T>C<br>*9:chr19:41512841G>T<br>*6 = *4+*9<br>*5:chr19:41522715C>T<br>*22:chr19:41522715C>T	CYP2C19<br>(90 208 bp)<br>SRS | LRS<br>*17:chr10:96521657C>T<br>*35:chr10:96535124A>G<br>*2:chr10:96541616G>A	CYP2C9<br>(51 136 bp)<br>SRS | LRS<br>*2:chr10:96702047C>T<br>*8:chr10:96702066G>A<br>*9:chr10:96708974A>G<br>*3:chr10:96741053A>C	CYP3A4<br>(27 217 bp)<br>SRS | LRS<br>*1B:chr7:99382096T>C<br>*22:chr7:99366316G>A	CYP3A5<br>(31 802 bp)<br>SRS | LRS<br>*1T,*3G:chr7:99270539C>T	DPYD<br>(843 316 bp)<br>SRS | LRS<br>chr1:97915614C>T<br>chr1:97981343A>C<br>chr1:98039419C>T<br>chr1:98045449G>C	TPMT<br>(26 858 bp)<br>SRS | LRS<br>rs1800460<br>rs1142345<br>rs1800462	UGT2B15<br>(24 179 bp)<br>SRS | LRS<br>*2:chr4:69536084C>A
Sample 1	*1/*1F	*1/*1	*1/*17	*1/*1	*1B/*1B	*1/*1	*1/*1	*1/*1	*1/*2
Sample 2	*1/*1F	*1/*4	*1/*1	*1/*1	*1/*1	*1/*3	*1/*1	*1/*1	*1/*2
Sample 3	*1F/*1F	*1/*1	*1/*1	*1/*1	*1/*22	*3/*3	*1/*1	*1/*1	*1/*2
Sample 5	*1F/*1F	*1/*6	*1/*1	*1/*2	*1/*1	*3/*3	*1/*1	*1/*1	*1/*2
Sample 6	*1F/*1F	*1/*1	*2/*2	*1/*1	*1/*1	*3/*3	*1/*1	*1/*1	*1/*2
Sample 7	*1F/*1F	*1/*1	*2/*2	*1/*1	*1/*1	*3/*3	*1/*1	*1/*1	*1/*2
Sample 8	1F/*1F	*1/*1	*2/*2	*1/*1	*1B/*1B	*1/*1	*1/*1	*1/*1	*1/*2
Sample 9	*1F/*1F	*1/*6	*1/*1	*8/*8	*1B/*1B	*1/*1	*1/*1	*1/*1	*1/*2
Sample 10	*1F/*1F	*1/*22	*1/*1	*1/*1	*1/*1	*3/*3	*1/*1	*1/*1	*1/*2
Sample 11	*1/*1F	*1/*6	*17/*17	*1/*1	*1/*1	*3/*3	*1/*1	*1/*1	*1/*2
Sample 12	*1F/*1F	*4/*6	*1/*2,*35	*1/*1	*1/*1	*1/*1	*1/*1	*1/*1	*1/*2
Sample 13	*1F/*1F	*1/*5	*1/*2,*35	*1/*1	*1/*1	*1/*1	*1/*1	*1/*1	*1/*1
